# Peripheral blood monocytes as a therapeutic target for marrow stromal cells in stroke patients

**DOI:** 10.3389/fneur.2022.958579

**Published:** 2022-10-05

**Authors:** Nikunj Satani, Kaushik Parsha, Courtney Davis, Adrian Gee, Scott D. Olson, Jaroslaw Aronowski, Sean I. Savitz

**Affiliations:** ^1^Department of Neurology, McGovern Medical School, Institute for Stroke and Cerebrovascular Diseases, The University of Texas Health Science Center at Houston, Houston, TX, United States; ^2^Center for Cell and Gene Therapy, Baylor College of Medicine, Houston, TX, United States; ^3^Department of Pediatric Surgery, McGovern Medical School at UTHealth, The University of Texas Health Science Center at Houston, Houston, TX, United States

**Keywords:** monocytes, mesenchymal stromal cells (MSCs), stromal cells, stroke, secretome

## Abstract

**Background:**

Systemic administration of marrow stromal cells **(MSCs)** leads to the release of a broad range of factors mediating recovery in rodent stroke models. The release of these factors could depend on the various cell types within the peripheral blood as they contact systemically administered MSCs. In this study, we assessed the immunomodulatory interactions of MSCs with peripheral blood derived monocytes **(Mϕ)** collected from acute stroke patients.

**Methods:**

Peripheral blood from stroke patients was collected at 5–7 days (*N* = 5) after symptom onset and from age-matched healthy controls (*N* = 5) using mononuclear cell preparation (CPT) tubes. After processing, plasma and other cellular fractions were removed, and Mϕ were isolated from the mononuclear fraction using CD14 microbeads. Mϕ were then either cultured alone or co-cultured with MSCs in a trans-well cell-culture system. Secretomes were analyzed after 24 h of co-cultures using a MAGPIX reader.

**Results:**

Our results show that there is a higher release of IFN-γ and IL-10 from monocytes isolated from peripheral blood at day 5–7 after stroke compared with monocytes from healthy controls. In trans-well co-cultures of MSCs and monocytes isolated from stroke patients, we found statistically significant increased levels of IL-4 and MCP-1, and decreased levels of IL-6, IL-1β, and TNF-α. Addition of MSCs to monocytes increased the secretions of Fractalkine, IL-6, and MCP-1, while the secretions of TNF-α decreased, as compared to the secretions from monocytes alone. When MSCs were added to monocytes from stroke patients, they decreased the levels of IL-1β, and increased the levels of IL-10 significantly more as compared to when they were added to monocytes from control patients.

**Conclusion:**

The systemic circulation of stroke patients may differentially interact with MSCs to release soluble factors integral to their paracrine mechanisms of benefit. Our study finds that the effect of MSCs on Mϕ is different on those derived from stroke patients blood as compared to healthy controls. These findings suggest immunomodulation of peripheral immune cells as a therapeutic target for MSCs in patients with acute stroke.

## Introduction

Mesenchymal stromal cells (MSCs) have shown promising results in preclinical studies for their therapeutic effects after ischemic stroke (1). Multiple clinical trials are examining the safety and potential efficacy of MSCs in stroke patients (2, 3). A comprehensive meta-analysis and systematic review on all the preclinical studies over the past 20 years shows that intravenous delivery of MSCs leads to functional recovery after stroke (4). There has been accumulating evidence which suggests that MSCs predominantly exert their beneficial influence *via* paracrine and immunomodulatory mechanisms (5). MSCs release cytokines and growth factors capable of modulating the behavior of a broad number of target cells (immunocytes, microglia, neurons etc) (6–9), resulting in immunomodulation, decreased apoptosis, increased angiogenesis, synaptogenesis, and endogenous neural stem cell activation (10–12). Immune cells such as T and B lymphocytes, monocytes (Mφ), dendritic cells have been shown to be targets for MSC derived factors (13, 14). Membrane vesicles/microparticles (MVs) are released from MSCs and are considered to play a role in intercellular communication (15–17). Cargo carried by MVs is instrumental in modulating target cell responses (18, 19).

MSCs express TLR (Toll-like Receptors) family of receptors, which may confer upon them, an ability to respond to the local microenvironment, which influences the MSC response (20–22). Recent *in vitro* studies report that pre-conditioning MSCs with inflammatory factors increases the release of mechanistically relevant bioactive factors and enhances their efficacy (23–25). Upon systemic administration, peripheral blood is the first environment encountered by MSCs where they invariably interact with the various immune cell components of peripheral blood, including monocytes. In the immediate timing after the ischemic stroke event, there is an induction of a peripheral inflammatory response evidenced by an increase in peripheral pro-inflammatory cells and cytokines (26, 27). Blood-derived monocytes (Mφ) have been shown to play a pivotal role in inflammation, both at the onset of stroke, as well as to aggravate the stroke lesions (28, 29). In addition, circulating monocytes have the potential of differentiating into many different types of cells including macrophages, given the right conditions (30). It has been shown that ischemic stroke differentially regulates monocyte subsets, which directly affect ischemic stroke pathology and could affect stroke outcomes (28).

The aim of this study, therefore, was to investigate the effects of exposing MSCs to peripheral blood derived monocytes from stroke patients, as measured by the release of cytokines. Since the interaction between MSCs and circulating monocytes in stroke patients may be an important aspect of MSC induced immune modulation (31), we studied the effects of co-culturing monocytes, isolated from healthy controls and stroke patients, and MSCs, on the cytokine release profiles from MSCs. We also observed the percentage change between secretome release from co-cultures of MSCs and monocytes vs. that from monocytes alone.

## Materials and methods

MSCs were derived and expanded from commercially available fresh whole bone marrow (AllCells) acquired from a single, healthy 34 year old male as previously published and cultured using standard tissue culture plastic in a media containing lot selected FBS (Altanta Bio) (32–34). Freshly thawed and washed BM-MSCs were used in all experiments of this study. MSCs used in this study met the criteria of MSC as outlined by the ISCT position paper (35), including ≥95% expression of CD73 and CD90 with ≤ 2% expression of CD45, CD34, CD11b, CD19, and HLA-DR [see Supplementary Table 2 from (32)].

To replicate the conditions of a clinical trial involving intravenous MSC administration in stroke patients, we exposed MSCs to peripheral blood monocytes derived from patients with acute ischemic stroke. Anti-coagulated (heparin) peripheral blood was collected from age-matched healthy controls (*n* = 5) and stroke patients (*n* = 5) in a vacutainer CPT tube (BD Biosciences). The age, gender and NIHSS of enrolled stroke patients and healthy controls are detailed in Supplementary Table 1. 10 mls of peripheral blood was drawn once at day 5–7 after stroke onset. The blood was spun at 1800xg for 20 min and the mononuclear fraction was collected from CPT tube. Peripheral monocytes from the blood of healthy and stroke subjects was isolated using MACS isolation kit (Miltenyi Biotech, #130-091-153) from the mononuclear fraction of CPT tube. Co-culture assays were conducted by placement of 100,000 cells, in 250 μl Plasma free media, in the top chamber of a 0.8 μm trans-well system (BD Falcon,NJ) or into a regular well of a 24 well plate. The same number of MSCs suspended in 250 μl of Plasma free media, were placed in the bottom chamber of the trans-well or directly into the regular well of the 24 well plate containing the monocytes. The 2-chamber assay provided for a free exchange of media along with the secretomes from MSCs and monocytes, between the top and bottom chamber. To serve as a baseline control, each monocyte sample was placed in Plasma free media alone for 24 h. Additionally, MSCs were also placed alone for 24 h in Plasma free media. At the end of 24 h the media was collected, centrifuged at 800 g for 4 mins in a table top centrifuge and cell free supernatant collected and stored at −80°C. Cytokine analysis was performed on the stored samples using a MAGPIX magnetic bead based ELISA assay (Millipore) to test for IL-4, IL-6, TNF-α, IL-1β, IL-10, IFN-γ, MCP-1 and Fractalkine.

Statistical analysis was performed using non-parametric Matt-Whitney test for each secretome and between co-culture groups from healthy control and stroke patients. Each group had a sample size of *N* = 5. The *p*-value of < 0.05 was considered significant. Error bars represent standard error of mean (SEM).

## Results

### Secretome release from monocytes isolated from stroke patients

**Figure 1:**
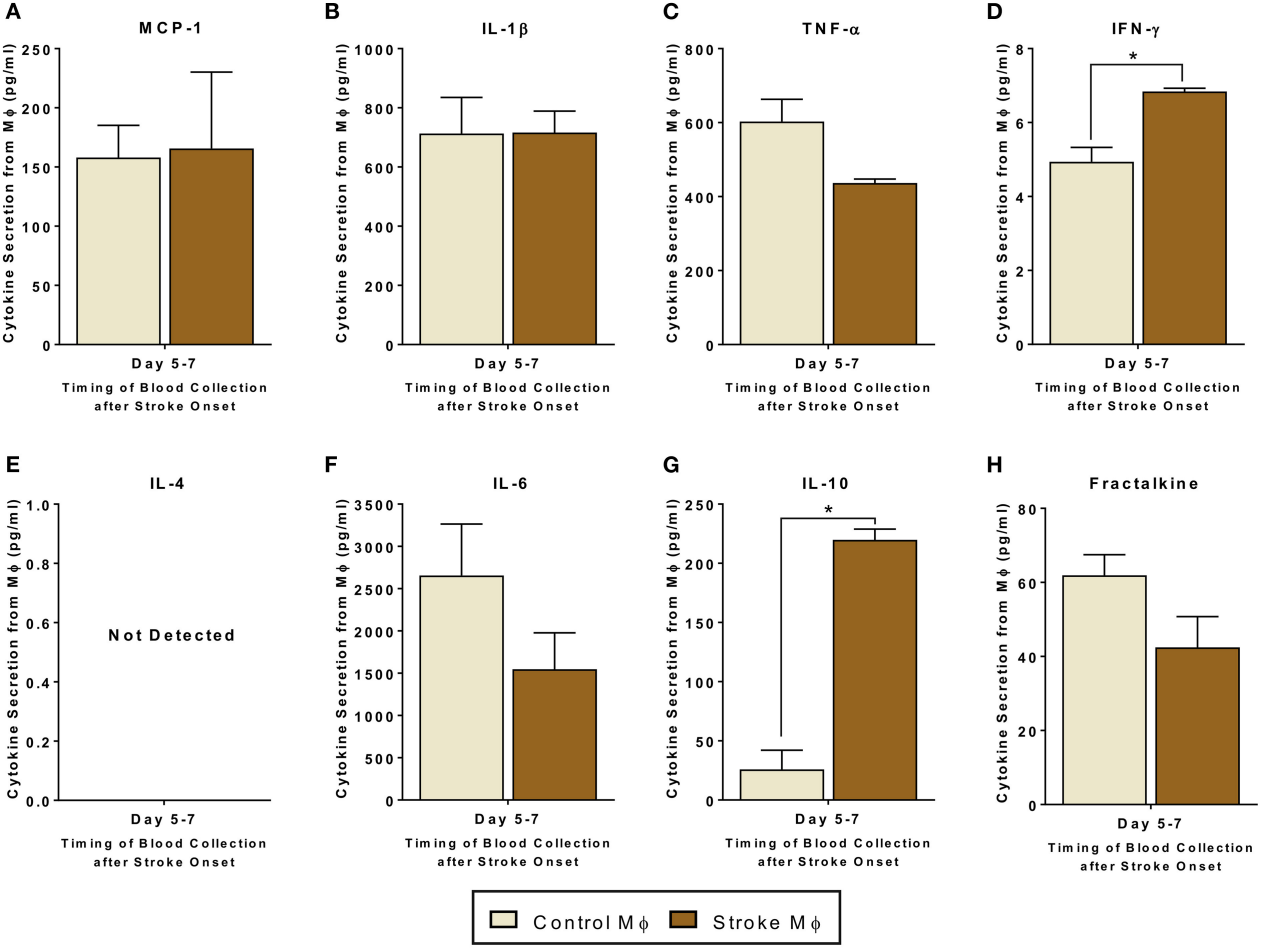
Secretomes released from peripheral blood monocytes (Mϕ). **(A–H)** represents secretions of MCP-1, IL-1β, TNF-α, IFN-γ, IL-4, IL-6, IL-10, and Fractalkine. IL-4 secretions were not detectable. Monocytes are isolated from peripheral blood of healthy controls and stroke patients, and cultured alone for 24 h (*N* = 5, error bars represent SEM, *p < 0.05).

Before studying the co-cultures and trans-well cultures of monocytes and MSCs, we studied the secretome from monocytes from both healthy control and stroke patients. In monocytes isolated from stroke patients, we found significantly higher release of IFN-γ and IL-10 as compared to that from monocytes isolated from healthy controls (Figure 1, *p* < 0.05). Even though there was a trend of reduced TNF-α and Fractalkine release from monocytes of stroke patients, this trend was not statistically significant (Figure 1C, Supplementary Table 2).

### Secretome changes from co-cultures of peripheral monocytes from stroke patients with MSCs

**Figure 2:**
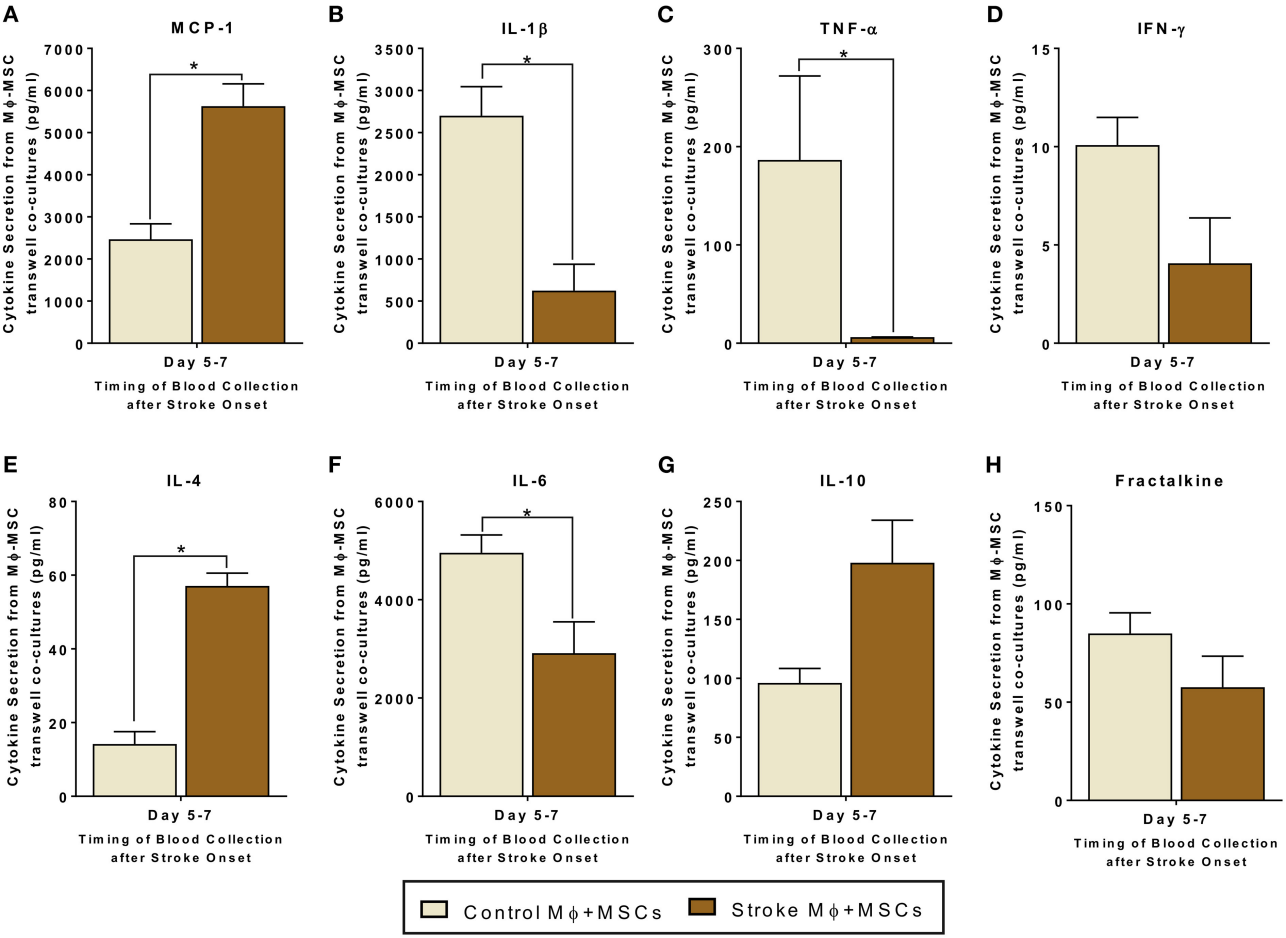
Secretomes released from trans-well co-cultures of peripheral blood monocytes (Mϕ) and human bone marrow derived mesenchymal stromal cells (MSCs). **(A–H)** represents secretions of MCP-1, IL-1β, TNF-α, IFN-γ, IL-4, IL-6, IL-10, and Fractalkine. Monocytes are isolated from peripheral blood of healthy controls and stroke patients, and cultured with MSCs for 24 h (*N* = 5, error bars represent SEM, *p < 0.05).

**Figure 3:**
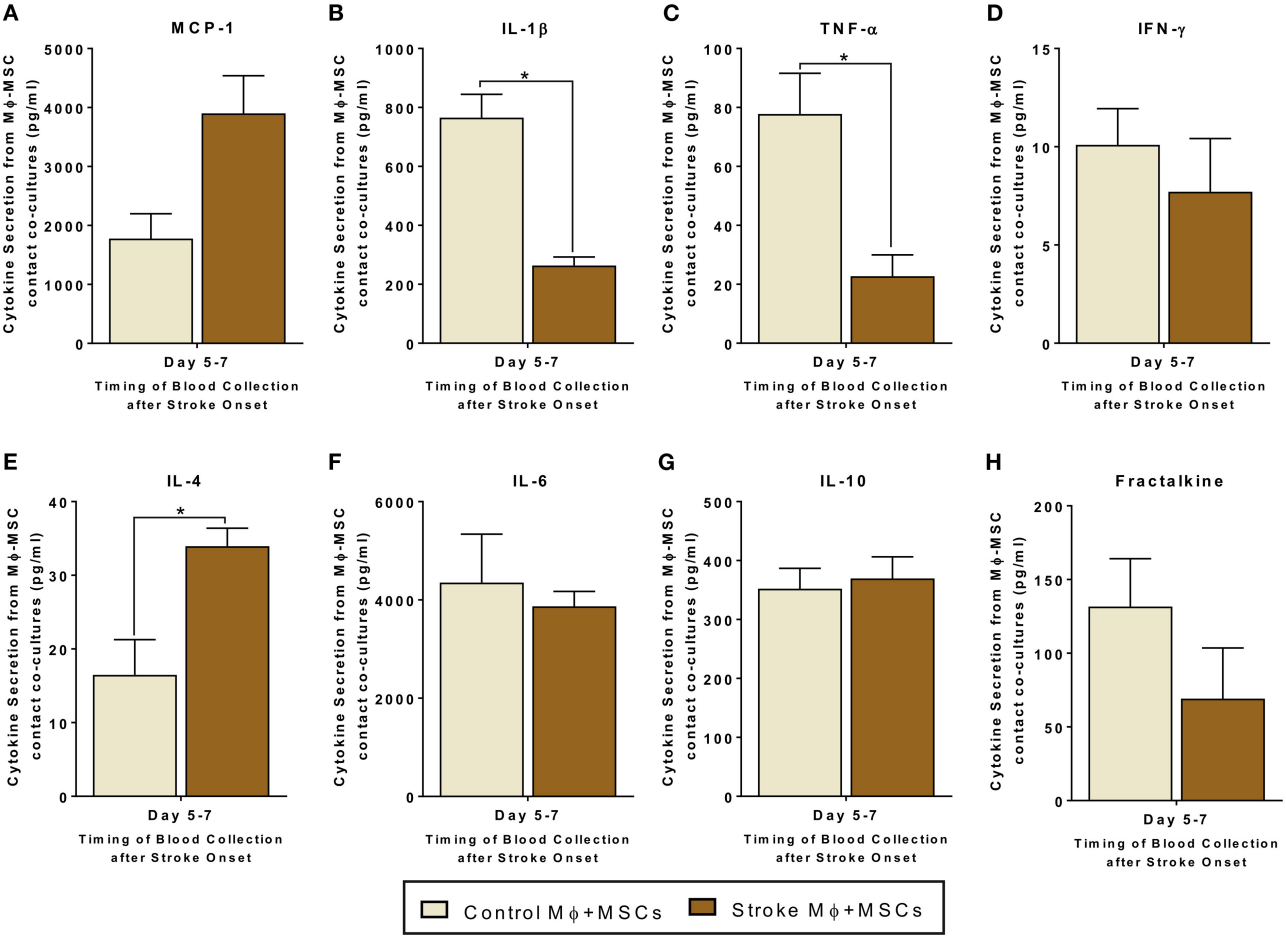
Secretomes released from contact co-cultures of peripheral blood monocytes (Mϕ) and human bone marrow derived mesenchymal stromal cells (MSCs). **(A–H)** represents secretions of MCP-1, IL-1β, TNF-α, IFN-γ, IL-4, IL-6, IL-10, and Fractalkine. Monocytes are isolated from peripheral blood of healthy controls and stroke patients, and cultured with MSCs for 24 h (*N* = 5, error bars represent SEM, *p < 0.05).

Since monocytes are one target of MSCs in the circulation, we examined the interactions of monocytes and MSCs after stroke. We studied both trans-well co-cultures as well as contact co-cultures to study direct interactions between monocytes and MSCs, as well as indirect interactions between them *via* release of secretomes (Figures 2, 3).

For trans-well co-cultures involving monocytes isolated from stroke blood, there was a significantly higher release of MCP-1 and IL-4, as compared to co-cultures using monocytes from healthy controls (Figures 2A,E, *p* < 0.05). There was also a statistically significant decrease in the release of IL-6, IL-1β, and TNF-α from trans-well co-cultures of MSCs with stroke derived monocytes as compared to co-cultures using healthy monocytes (Figures 2C,H, *p* < 0.05). Furthermore, IL-10 showed a trend of increased secretion, and IFN-γ showed a trend of decreased secretion from trans-well co-cultures involving stroke monocytes, however this trend did not reach significance (Figures 2D,G, Supplementary Table 2).

Contact co-cultures showed similar trends in secretome release after MSC-Monocytes co-cultures (Figure 3). In contact co-cultures involving monocytes isolated from stroke blood, there was a statistically significant increased release of IL-4 as compared to co-cultures involving healthy control monocytes (Figure 3E, *p* < 0.05). There was also a decrease in the release of IL-1β and TNF-α from co-cultures involving monocytes from stroke patients (Figure 3C, *p* < 0.05). Similar to the trans-well co-cultures, MCP-1 showed a trend of higher secretion in contact co-cultures using stroke patient derived monocytes. However, this trend failed to reach statistical significance (Figure 3A, Supplementary Table 2).

In summary, for co-cultures involving monocytes isolated from stroke blood, we found significantly lower levels of pro-inflammatory secretome and high levels of anti-inflammatory secretome.

### Percentage change between secretome released from MSC-Mφ co-cultures and Mφ alone

To ascertain the secretome change caused due to addition of MSCs, we calculated the percentage change between secretome released from MSC-Mφ co-cultures and Mφ alone. Our results show that there was a large percentage increase in the release of Fractalkine (37.3%), IL-10 (270.8%), IL-1β (278.8%), and MCP-1 (1462.2%), and a large percentage decrease in the release of TNF-α (69.1%) when MSCs were co-cultured with Mφ from healthy controls in a trans-well system (Table 1). This pattern was true for contact co-cultures as well.

**Table 1:** Percentage change between secretome released from MSC-Mϕ co-cultures and Mϕ alone.

	**Percentage change between**	**Percentage change between**	**Percentage change between**	**Percentage change between**
	**trans-well co–cultures**	**trans-well co–cultures of**	**contact co–cultures of**	**contact co–cultures of**
	**of healthy control Mϕ**	**stroke patients Mϕ and**	**healthy control Mϕ**	**stroke patients Mϕ**
	**and MSCs vs. Mϕ alone**	**MSCs vs. Mϕ alone**	**and MSCs vs. Mϕ alone**	**and MSCs vs. Mϕ alone**
**Fractalkine**	37.3	35.7	112.6	62.9
IFN–γ	104.1	−40.5	104.4	12.7
IL−10	270.8	−9.9	1,258.1	68.2
IL−1β	278.8	−12.9	7.4	−63.2
IL−4	Not detected in Mϕ alone groups
IL−6	86.7	88.1	64.0	150.0
MCP−1	1,462.2	3,296.0	1,024.2	2,254.6
TNF–α	−69.1	−98.6	−87.1	−94.8

Percentage change was not calculated for IL−4 because IL−4 was not detected in Mϕ alone groups.

Interestingly, there was a decrease in percentage change of IFN-γ when stroke monocytes were involved in the co-cultures as compared to healthy control monocytes (Table 1). Also, IL-1β, IFN-γ, and TNF-α showed greater percentage decrease when stroke monocytes were involved in both trans-well and contact co-cultures, as compared to when healthy control monocytes were involved.

## Discussion

More than 20 years of research indicates that intravenously delivered MSCs confers benefit in animal stroke models but MSCs do not enter into the brain but remain in peripheral tissues (4, 36–38). MSCs release multiple bioactive factors, such as cytokines and MVs, which may contribute to the underlying beneficial effects in stroke and other disease models (39–42). Acute stroke induces an initial state of systemic inflammation characterized by an increase in the levels of circulating pro-inflammatory immune cells. The cellular components of peripheral blood are therefore potential targets of how MSCs modulate the immune response to injury. For example, the interaction MSCs with monocytes in the peripheral circulation may play an important role in the downstream effects of MSCs. A previous study has already shown that monocytes are major players in prognosis and risk of infection after stroke (43). The study also showed that the total number of monocytes in circulation increase around day 2 after stroke onset, but reach highest number around day 7 after stroke onset. Hence, we decided to study the interaction of MSCs with peripheral monocytes derived from stroke patients at day 5–7 after stroke onset.

Direct or trans-well crosstalk between MSCs and monocytes led to an increase in anti-inflammatory and a decrease in pro-inflammatory cytokine release. Of particular note, our data show that there is a significant decrease in the release of IL-1β and TNF-α from co-cultures of peripheral blood monocytes and MSCs (Figures 2, 3). Similarly, our data also show a significant increase in the release of IL-4 from co-cultures of peripheral blood monocytes and MSCs (Figures 2, 3). MCP-1 secretions are significantly increased in trans-well co-cultures, while in contact co-cultures they show a trend of increase, even though they fail to reach significance statistically. The lack of substantial differences between the results of trans-well vs. co-culture suggest that MSCs do not have to make contact with monocytes. MSCs may release factors that directly affect monocytes and those interactions may lead to an amplification or suppression of different types of cytokine release, in favor of an anti-inflammatory environment.

Our results also suggest that there is an increase in the release of Fractalkine, IL-6 and MCP-1 when MSC and Mφ are co-cultured, as compared to Mφ alone (Table 1). We found that the secreted levels of IL-6 were increased in stroke patient derived Mφ-MSC co-cultures, far more than healthy control derived Mφ-MSC co-cultures.

On the other hand, IFN-γ, IL-1β, and TNF-α showed reduced secretions in Mφ-MSC co-cultures involving stroke patient derived Mφ, as compared to healthy control derived Mφ. These results are consistent regardless of trans-well or contact co-cultures. Stroke patient's blood is known to carry more inflammatory factors with the monocytes from stroke blood showing a more inflammatory phenotype (43). Monocytes have been shown in literature to be major players in the prognosis after stroke (43, 44). It is possible therefore, that systemic administration of MSCs in stroke patients will expose the administered cells to an inflammatory environment upon entering the circulation and the subsequent inevitable interaction between MSCs and the circulating factors may lead to their activation. Once activated, the cells might release therapeutically relevant factors that have been implicated in their efficacy in animal models. There have been reports of better behavioral outcomes with the use of MSCs derived from stroke induced rats compared to healthy rats (45). Indeed, this effect of ischemic pre-conditioning has been utilized in the design of a clinical trial, wherein, autologous MSCs cultured in plasma from stroke patients are being injected intravenously (46). Our results show that there is a possibility that interaction of MSCs with Mφ is quite different if the Mφ are derived from stroke patients' blood as compared to healthy control blood. This point to the possibility that inflamed monocytes could be behaving differently in presence of MSCs as compared to normal healthy monocytes. In fact, inflamed monocytes could be licensing the MSC function such that the pro-regenerative secretome is increased and inflammatory secretome is decreased. MSCs pre-treated with inflammatory mediators such as TNF-α, IL1β or Nitric Oxide (NO), release immunomodulatory cytokines in significantly greater amounts, possibly *via* their interaction with the TLR receptors on the MSCs (23–25). This may indicate that MSCs could be activated once they are exposed to certain mediators, that could be released from monocytes, and subsequently may release biological factors appropriate to the surrounding environment. Our results from co-culture system indicate that there is some interplay between MSCs and Mφ, which could be mediated by the trophic factors released by stroke patient derived Mφ. Further studies will be needed to ascertain the mechanisms involved in such licensing.

Previously, it has been shown that MSCs can inhibit the release of TNF-α and MIP-1β from non-classical monocytes derived from healthy blood, and thereby reduce the expression of pro-inflammatory cytokines and chemotactic factors, which can ultimately reduce the development of an inflammatory immune response (47). Similarly, our study shows reduction in TNF-α from our co-culture experiments. In addition, we have previously studied the effects of various clinically relevant medications on Mφ-MSC co-cultures (48–50). However we have never reported the interactions of MSCs with stroke derived as well as healthy control Mφ. Our study provides first such insight into an interaction that could be useful in designing clinical trials in future. Indeed, in a recently published STEPS (Stem Cells as an Emerging Paradigm in Stroke) guidelines, it is extensively discussed how important it is to study the drug-cell interactions, cell-cell interactions and develop other validated potency assays (51). This study indicates that investigating cell-to-cell interaction should be of paramount importance before designing clinical trials; as such interactions could be pivotal to the success of clinical trials. To our knowledge, this is the first study to provide data suggesting direct crosstalk between monocytes and MSCs. Previous time course studies have observed that TNF-α levels in monocytes derived from stroke patients peaks at around 72 h after stroke onset, and then decrease at day 7 after stroke onset (52). This could also indicate a change in the composition of monocyte subtypes that can be studied further in more thorough future time course studies. One of the limitations of our study is that in co-culture experiments, it was not possible to separate Mφ and MSCs after the experiment, because of the fragile nature of monocytes. Due to this limitation, we could not determine if the changes we observe derive from Mφ, MSCs, or both. Nonetheless, the results themselves are clinically relevant for MSC based clinical trials in stroke, where MSCs will interact with Mφ after entering patients' blood.

In conclusion, our data suggest that upon introduction into the peripheral circulation, MSCs may interact with monocytes in the peripheral circulation, which leads to immunomodatory changes in a number of different cytokines, favoring an anti-inflammatory environment. These results that have implications to define key mechanisms of action for the systemic delivery of MSCs in acute stroke.

## Data availability statement

The original contributions presented in the study are included in the article/Supplementary material, further inquiries can be directed to the corresponding author.

## Author contributions

NS wrote the manuscript. NS and CD conducted the statistical analysis and wrote the statistical sections in manuscript. NS, KP, and CD collected the data. NS and KP designed the study. AG and SO provided the clinical grade human bone marrow mesenchymal stromal cells for the study. NS, JA, and SS oversaw the project and edited the manuscript. All authors contributed to the article and approved the submitted version.

## Conflict of interest

The authors declare that the research was conducted in the absence of any commercial or financial relationships that could be construed as a potential conflict of interest.

## Publisher's note

All claims expressed in this article are solely those of the authors and do not necessarily represent those of their affiliated organizations, or those of the publisher, the editors and the reviewers. Any product that may be evaluated in this article, or claim that may be made by its manufacturer, is not guaranteed or endorsed by the publisher.
