# Supplementary material for: Peripheral blood monocytes as a therapeutic target for marrow stromal cells in stroke patients
Source: Front Neurol. 2022 Oct 5;13:958579. doi: 10.3389/fneur.2022.958579 (PMC9580494; doi:10.3389/fneur.2022.958579)
Supplement: Supplementary file 1 [file Data_Sheet_1.PDF]

SUPPLEMENTARY DATA

Peripheral Blood Monocytes as a Therapeutic Target for Marrow Stromal Cells in Stroke Patients

Nikunj Satani[1*], Kaushik Parsha[1], Courtney Davis[1], Adrian Gee[2], Scott D. Olson[3], Jaroslaw Aronowski[1], Sean I. Savitz[1]

[1]Institute for Stroke and Cerebrovascular Diseases, The University of Texas Health Science Center at Houston, McGovern Medical School, Houston TX 77030

[2]Center for Cell and Gene Therapy, Baylor College of Medicine, Houston, TX.

[3]Department of Pediatric Surgery, The University of Texas Health Science Center at Houston, McGovern Medical School at UTHealth, Houston, Texas, USA

Supplementary Table 1. Age, Gender and NIHSS for subjects enrolled in this study.

	**Age**	**Gender**	**NIHSS**
**Control 1**	62	M	-
**Control 2**	58	M	-
**Control 3**	77	M	-
**Control 4**	61	F	-
**Control 5**	44	M	-
**Stroke Patient 1**	83	F	13
**Stroke Patient 2**	59	M	9
**Stroke Patient 3**	60	M	13
**Stroke Patient 4**	72	M	15
**Stroke Patient 5**	77	M	9
**Mann-Whitney test for Age between Controls vs Stroke Patients**			**p-value =0.34**

Supplementary Table 2. Significance values (p-values) for the monocyte, trans-well as well as contact co-cultures. Matt-Whitney test was used to analyze the results. p-value of less than 0.05 was considered significant. N=5

	**Control vs Stroke Mϕ cultured alone**	**Control Mϕ-MSC transwell co-cultures vs Stroke Mϕ-MSC transwell co-cultures**	**Control Mϕ-MSC contact co-cultures vs Stroke Mϕ-MSC contact co-cultures**
**Fractalkine**	0.09	0.42	0.09
**IFN-γ**	< 0.05	0.09	0.42
**IL-10**	< 0.05	0.09	0.84
**IL-1β**	> 0.99	< 0.05	< 0.05
**IL-4**	> 0.99	< 0.05	< 0.05
**IL-6**	0.15	< 0.05	0.42
**MCP-1**	> 0.99	< 0.05	0.09
**TNF-α**	0.09	< 0.05	< 0.05
